# Need for longitudinal evidence on child and adolescent nutrition in the Amazon

**DOI:** 10.1017/S1368980026102754

**Published:** 2026-05-28

**Authors:** Gelner Archenti, Haydeé Cárdenas-Quintana

**Affiliations:** 1 Doctorado en Nutrición – Escuela de Posgrado, Universidad Nacional Agraria La Molina, Lima, Peru; 2 Instituto de Seguridad Alimentaria y Nutricional, Universidad Nacional Agraria La Molina, Lima, Peru

**Keywords:** Adolescent nutrition, Child nutrition, Amazon, Cohort studies

To the editor,

We read with great interest the recently published research by Martins da Costa and colleagues, where they evaluated overweight and obesity among children and adolescents in the Amazonian region of Rondônia, Brazil, over almost 20 years^(1)^. We are currently working on a project evaluating the nutritional status of children in Alto Amazonas in the Peruvian region of Loreto, Peru. While the evaluation we are conducting is very comprehensive, funding and logistic concerns limited us to a cross-sectional design. A deep review of the literature let us to realise that most of the evidence from the South American Amazon similarly relies on cross-sectional studies like ours or repeated cross-sectional evaluations, such as the study by Martins da Costa^(1)^ or the Tsimane Health and Life History Project in Bolivia^(2)^.

Longitudinal evidence in Amazonian populations remains rather scarce, is geographically concentrated, often involves limited follow-up periods and frequently assesses a limited range of nutritional outcomes. To our knowledge, seven longitudinal studies have been conducted in the Amazon region^(3–9)^: four in Brazil^(3–6)^, one in Ecuador^(7)^ and two in Peru^(8,9)^. The majority focused on social determinants of health and nutrition^(3,5–7)^, whereas the other studies addressed topics like breast-feeding^(4)^, or the interrelation between nutrition and enteric infections^(8)^ and mercury exposure during pregnancy^(9)^. While the list is largely comprehensive, it may not be fully exhaustive of all existing cohorts among Amazonian populations. Other studies have been conducted across multiple regions within a country, such as the Young Lives Study in Peru – conducted in Amazonas and San Martín but not exclusively in the Amazon. While highly valuable, such studies include children from different geographical and cultural contexts and may overlook important nuances that are specific to populations living in the Amazon. Therefore, they have not been included in the table.

The scarcity of longitudinal evidence highlights the knowledge gap regarding the health of Amazonian populations. The Amazon region extends across Brazil, Peru, Colombia, Bolivia, Ecuador, Venezuela and the Guianas and is home to 40 million people, including more than 300 ethnic groups of Amazonian Indigenous peoples^(10)^. Many of these populations experience poverty, with limited access to basic needs, with food and water insecurity, and exposed to multiple health threats^(2–8,10)^. The scarcity of longitudinal evidence prevents a deeper understanding of changes in nutritional status over time and limits the ability to identify causal determinants of disease in these particular populations.

It is important to note that numerous logistical challenges complicate the conduct and implementation of longitudinal studies of this nature, including geographical inaccessibility, sparse populations and linguistic and cultural barriers. Moreover, these populations are particularly vulnerable, and research must strictly adhere to country-specific legislation, including assessments from Indigenous authorities and research ethics committees.

We would like to use this platform to make a call on researchers in the Amazon to, where feasible, prioritise the design and establishment of long-term longitudinal studies that can generate highly valuable evidence. We would further argue that there is scope for the development of multi-centre Amazonian cohorts spanning several countries – ideally with multi-country funding – as populations living across the Amazon basin may share important similarities across certain social, environmental and structural dimensions of life, while simultaneously exhibiting substantial ethnic, cultural and linguistic diversity. This combination of shared contexts and heterogeneity, further interacting with different levels of urbanisation and adoption of Western-like practices, is likely to result in complex but potentially region-specific causal mechanisms for nutrition and health outcomes among children and adolescents. Longitudinal research is essential to disentangle these mechanisms and to inform effective, context-specific action to improve child and adolescent nutrition in the Amazon.
